# The Neural Dynamics of Facial Identity Processing: Insights from EEG-Based Pattern Analysis and Image Reconstruction

**DOI:** 10.1523/ENEURO.0358-17.2018

**Published:** 2018-02-26

**Authors:** Dan Nemrodov, Matthias Niemeier, Ashutosh Patel, Adrian Nestor

**Affiliations:** 1 Department of Psychology, University of Toronto Scarborough, 1265 Military Trail, Toronto, Ontario M1C1A4, Canada

**Keywords:** ERP, face space, image reconstruction, N170, pattern analysis, spatiotemporal dynamics

## Abstract

Uncovering the neural dynamics of facial identity processing along with its representational basis outlines a major endeavor in the study of visual processing. To this end, here, we record human electroencephalography (EEG) data associated with viewing face stimuli; then, we exploit spatiotemporal EEG information to determine the neural correlates of facial identity representations and to reconstruct the appearance of the corresponding stimuli. Our findings indicate that multiple temporal intervals support: facial identity classification, face space estimation, visual feature extraction and image reconstruction. In particular, we note that both classification and reconstruction accuracy peak in the proximity of the N170 component. Further, aggregate data from a larger interval (50–650 ms after stimulus onset) support robust reconstruction results, consistent with the availability of distinct visual information over time. Thus, theoretically, our findings shed light on the time course of face processing while, methodologically, they demonstrate the feasibility of EEG-based image reconstruction.

## Significance Statement

Identifying a face is achieved through fast and efficient processing of visual information. Here, we investigate the nature of this information, its specific content and its availability at a fine-grained temporal scale. Notably, we provide a way to extract, to assess and to visualize such information from neural data associated with individual face processing. Thus, the present work accounts for the time course of face individuation through appeal to its underlying visual representations while, also, it provides a first demonstration regarding the ability to reconstruct the appearance of stimulus images from electroencephalography (EEG) data.

## Introduction

Elucidating the dynamics of visual face processing is essential to understanding its underlying mechanisms. To this end, considerable efforts have been devoted to characterizing the time course of face processing especially through the use of electroencephalography (EEG) and magnetoencephalography (MEG) given the temporal resolution of these methods. Accordingly, much is known about the temporal profile of face processing as reflected by either traditional event-related potentials (ERPs; Bentin and Deouell, 2000; Itier and Taylor, 2002; Huddy et al., 2003; Tanaka et al., 2006; Rossion and Caharel, 2011; Zheng et al., 2012), or by spatiotemporal patterns (Liu et al., 2009; Cichy et al., 2014; Vida et al., 2017). Comparatively less is known about the visual representations underlying the dynamics of face processing, especially as related to facial identity. To shed light on this issue, the present work employs an image-reconstruction paradigm (Miyawaki et al., 2008; Naselaris et al., 2009; Nishimoto et al., 2011; Cowen et al., 2014; Chang and Tsao, 2017) seeking to approximate the visual appearance of individual faces from spatiotemporal EEG patterns. Concretely, this work aims to answer whether the visual information involved in face identification can be recovered from EEG signals and, further, whether such information can support the characterization of neural-based face space along with the reconstruction of individual face images.

Recent applications of pattern analysis have focused on the temporal profile of face discrimination at the category (Carlson et al., 2013; Van de Nieuwenhuijzen et al., 2013; Cauchoix et al., 2014; Cichy et al., 2014; Kaneshiro et al., 2015) and the exemplar level (Davidesco et al., 2014; Ghuman, et al., 2014). For instance, expression-invariant identity discrimination has been conducted using MEG (Vida et al., 2017), electrocorticography (Ghuman et al., 2014) and EEG (Nemrodov et al., 2016). These studies found multiple, distinct temporal windows sensitive to facial information and, consistent with results from monkey neurophysiology (Hung et al., 2005; Freiwald et al., 2009) and human psychophysics (Lehky, 2000; Tanaka and Curran, 2001; Crouzet et al., 2010), have estimated an early onset for such sensitivity (Ghuman et al., 2014; Nemrodov et al., 2016). Further, the cortical source of this information was attributed primarily to the fusiform gyrus (FG) in line with homologous investigations of face identification using functional magnetic resonance imaging (fMRI; Nestor et al., 2011; Goesaert and Op de Beeck, 2013; Anzellotti et al., 2014).

Yet, the representational basis of facial identity that allows successful discrimination from neural data remains to be elucidated. Arguably, neural patterns elicited by face perception speak to the properties of a representational face space (Valentine, 1991), or, more generally, of a representational similarity space (Kriegeskorte et al., 2008). In an effort to clarify the nature of such representations, recent fMRI work (Nestor et al., 2016) has combined the study of face space and neural-based image reconstruction. Specifically, this work has derived visual features from the structure of FG-based face space and, then, used such features for facial image reconstruction. However, this work did not consider the temporal aspects of face processing, neither did it assess the invariant structure of a facial identity space, surprisingly, we note that face space invariance over common image transformations (e.g., viewpoint, expression) has rarely been explicitly investigated (but see Newell et al., 1999; Blank and Yovel, 2011).

To address the issues above, the current work aims to derive face space constructs from the EEG signal associated with consecutive time windows separately for different facial expressions (i.e., neutral and happy). Then, it reconstructs the appearance of one set of faces (e.g., happy) based on the structure of the face space derived for the other faces (e.g., neutral). This demonstration provides evidence that the spatiotemporal information of EEG patterns is rich enough to support: (1) identity-level face discrimination; (2) neural-based face space estimation; (3) visual feature synthesis; and (4) facial image reconstruction. Further, this work characterizes the neural dynamics of expression-invariant face processing while, more generally, it provides proof of concept for the possibility of EEG-based image reconstruction.

## Materials and Methods

### Participants

Thirteen healthy adults (six males, seven females; age range: 18–27 years) with normal or corrected-to-normal vision were recruited from the University of Toronto community to participate in the study in exchange for monetary compensation. All participants provided informed consent and all experimental procedures were approved by the Research Ethics Board at the University of Toronto. The data of all participants were included in the analyses reported below.

### Stimuli

A total of 140 face images of 70 individuals, each displaying a neutral and a happy expression were used as experimental stimuli. Out of these, 108 images of 54 unfamiliar males were selected from three databases: AR (Martinez and Benavente, 1998), Radboud (Langner et al., 2010), and FEI (Thomaz and Giraldi, 2010). The remaining 32 images displayed faces of six famous male and 10 female individuals selected from open access sources. To be clear, unfamiliar male face stimuli are the focus of the present investigation while female faces were used as go trials in a go/no-go gender recognition task (see Experimental design) and additional famous male faces were included to promote alertness (however, no results are reported for them below due to the smaller stimulus set that precluded a separate examination of famous face recognition).

All images featured young adult white individuals with frontal view, gaze and illumination. The stimuli were selected so that no facial accessories, hair or makeup obscured the internal features of the face and so that all happy expressions displayed an open-mouth smile. These images were: (1) scaled uniformly and aligned with roughly the same position of the eyes and the nose; (2) cropped to eliminate background; (3) normalized with the same mean and root mean square (RMS) contrast values separately for each color channel in CIEL*a*b* color space; and (4) reduced to the same size (95 × 64 pixels).

### Experimental procedure

Before EEG testing participants were administered the Cambridge Face Memory Test (CFMT), (Duchaine and Nakayama, 2006) to confirm that their face processing abilities fall within the range of normal performance for young adults (Bowles et al., 2009). Participants also completed the Vividness of Visual Imagery Questionnaire 2 (VVIQ-2; Marks, 1995) along with a custom familiarity-rating famous face questionnaire.

During EEG sessions participants were seated in a dimly lit room at a viewing distance of 80 cm from an LCD monitor (resolution: 1920 × 1080, refresh rate: 60 Hz). The participants were instructed to perform a go/no-go gender recognition task by pressing a designated key every time they saw a female face, irrespective of expression. The experiment consisted of 32 blocks of stimulus presentation divided into two sessions conducted on different days. In each session, experimental blocks were preceded by one training block, subsequently discarded from all analyses. The blocks were separated by self-paced breaks.

Over the course of any given block, each image of a male face was presented twice and each image of a female face was presented once, for a total of 260 trials. Images were presented in a pseudorandom order under the constraint that no facial identity would appear consecutively. Each stimulus was presented in the center of the screen against a black background and subtended a visual angle of 3.2 × 4.9. A stimulus display lasted for 300 ms, and it was followed by a fixation cross for a duration ranging randomly between 925 and 1075 ms. Each session, including participant and equipment setup, lasted around 2.5 h. Stimulus presentation and response recording relied on Matlab 9.0 (Mathworks) and Psychtoolbox 3.0.8 (Brainard, 1997; Pelli, 1997).

### EEG acquisition and preprocessing

EEG data were recorded using a 64-electrode Biosemi ActiveTwo EEG recording system (Biosemi B.V.). The electrodes were arranged according to the International 10/20 System. The electrode offset was kept below 40 mV. The EEG and EOG were low-pass filtered using a fifth order sinc filter with a half-power cutoff at 204.8 Hz and then digitized at 512 Hz with 24 bits of resolution. All data were digitally filtered offline (zero-phase 24 dB/octave Butterworth filter) with a bandpass of 0.1–40 Hz.

Next, data were separated into epochs, from 100 ms before stimulus presentation until 900 ms later, and baseline corrected. Epochs corresponding to go trials (i.e., female face stimuli) and epochs containing false alarms were discarded from further analysis. Further, noisy electrodes were interpolated if necessary (no more than two electrodes per subject) and epochs were rereferenced to the average reference. In addition, before univariate ERP analysis, data were cleaned of ocular artifacts using Infomax ICA (Delorme et al., 2007).

After removing trials containing artifacts and/or false alarms, an average of 96% of trials (range: 75–100% across participants) were selected for further analysis. In particular, we note that relatively few trials contained false alarms as participants performed the go/no-go recognition task at ceiling (accuracy range: 98.1–100% across participants).

All analyses were conducted using Letswave 6 (http://nocions.webnode.com/letswave; Mouraux and Iannetti, 2008), MATLAB 9.0 and the G*Power toolbox (Faul, et al., 2007).

### Univariate ERP analyses

Twelve electrodes situated over homolog occipitotemporal areas (P5, P7, P9, PO3, PO7, and O1 on the left and P6, P8, P10, PO4, PO8, and O2 on the right) were selected for ERP analysis. Their selection was motivated by the relevance of these electrodes for face processing as revealed by ERP analysis (e.g., robust N170 amplitudes). Data from electrodes over the left and right sites were then averaged separately creating two sets of signals, one for each hemisphere.

For the purpose of univariate tests, the data were averaged for each unfamiliar face identity across expressions. Then, P1, N170 and N250 components were visually identified on a grand-average plot and a two-way repeated measures ANOVA over facial identities and hemispheres was conducted on maximum amplitudes in the 70–180 ms range for P1, and on minimum amplitudes in the 160–250 and 260–350 ms ranges for N170 and N250, respectively. Greenhouse–Geisser correction was applied in case of violation of the sphericity assumption.

### Pattern classification analysis

Epochs were linearly detrended, *z*-scored across time and electrodes, and corrected for outliers (i.e., values exceeding 3SD from the mean were thresholded at ±3 to minimize the deleterious effect of extreme values on SVM-based pattern classification). Then, all epochs were normalized to the same range (0–1) and mean (i.e., 0.5). To boost the signal-to-noise ratio (SNR) of spatiotemporal patterns (Grootswagers et al., 2017) multiple epochs pertaining to the same condition were averaged into ERP traces. Specifically, all epochs corresponding to the same image stimulus across two consecutive blocks, for a maximum of four epochs, were averaged together resulting in 16 separate traces per stimulus. Further, this procedure was also instrumental in handling missing data following trial removal and in balancing the number of observations for pattern classification. Specifically, since it is possible that both trials associated with a given stimulus in a given block be removed (e.g., due to artifacts), averaging data across single blocks can lead to different numbers of observations for different stimuli. However, averaging data across pairs of consecutive blocks (i.e., one to four trials per stimulus following data removal) ensured that equal numbers of observations can be constructed for each pair of stimuli undergoing pattern classification.

Next, to increase the robustness of pattern analyses epochs were divided into temporal windows containing five consecutive bins (5 bins × 1.95 ms ≈ 10 ms; Blankertz et al., 2011). For each window, data were concatenated into observations, for instance, data across selected occipitotemporal electrodes (see univariate ERP analyses) were concatenated into 60-dimension observations (5 time bins × 12 electrodes). These observations were constructed for the purpose of pattern analyses in time, window by window. In addition, we constructed more inclusive observations that contain all time bins between 50 and 650 ms after stimulus presentation (3684-dimension vectors: 307 bins × 12 electrodes), and, thus, both early and late information relevant for face processing (Ghuman et al., 2014; Vida et al., 2017). These higher-dimensional observations were constructed for the purpose of temporally cumulative analyses able to exploit more extensive information over time.

Pairwise discrimination of facial identity was conducted with the aid of linear support vector machine (SVM) classification (c = 1; SVMLIB 3.22; Chang and Lin, 2011) and leave-one-out cross-validation (i.e., one out of 16 pairs of observations was systematically left out for testing while the remaining 15 were used for training). Concretely, 1431 pairs of stimuli were classified across 16 leave-one-out cross-validation iterations and then classification accuracy was obtained for each pair by averaging across these iterations. Classification was conducted for all pairs of facial identities in two ways: (1) within expression, the classifier was trained separately on one expression, happy or neutral, and tested on the same expression; and (2) across expression, the classifier was trained on one expression and tested on the other. Significance of classification accuracy was assessed via a permutation test, by randomly shuffling identity labels 10^3^ times, and correcting for multiple comparisons across temporal windows using the false discovery rate (FDR).

The analyses above were conducted for data averaged across all participants (i.e., observations were constructed from averaged ERP traces). In addition, similar discrimination analyses were performed for data from individual participants. The significance of the overall classification accuracy for this analysis was assessed using one-sample two-tailed *t* tests against chance across participants separately for within- and across-expression discrimination.

Further, given that the three face databases used here for the purpose of stimulus selection and design sample different populations (i.e., individuals of different nationalities) we anticipated a possible effect of database. However, it is important to establish that any effects found here are not solely due to such differences. To assess this possibility, we also examined pairwise classification results for faces extracted from the same databases and, separately, for faces extracted from different databases, i.e., each face is classified only relative to faces from the same database, for a total of 472 pairs, or only relative to faces from different databases, for a total of 1918 pairs.

### Relationship with behavioral performance

Average pairwise face discrimination, as indexed by temporally cumulative analysis, was correlated with behavioral markers of performance (from CFMT and VVIQ-2 tests) for each participant.

For a finer-grained analysis in the temporal domain, pairwise face discrimination was correlated, window by window, with behavioral data from a previous study (Nestor et al., 2013, experiment 1) in which participants rated the visual similarity of pairs of faces. Specifically, in this study participants viewed a larger set of faces, including the 108 unfamiliar face images used here, and rated the similarity of each face with every other face on a five-point scale across a change in expression (i.e., one face in a pair displayed a neutral expression and the other a happy expression). Ratings from 22 healthy adult participants were then averaged to deliver an estimate of behavioral face similarity. Here, this estimate was compared to its neural counterpart from across-expression classification conducted on group-averaged ERP traces. Concretely, behavioral and neural-based estimates were related via Pearson’s correlation and tested for significance via a permutation test (10^3^ permutations for each temporal window; FDR correction across windows).

### Estimation of EEG-based face space

Face space constructs were derived by applying multidimensional scaling (MDS) to EEG-based estimates of face discrimination. Specifically, classification accuracies for pairs of facial identities were organized into a dissimilarity matrix in which each cell estimates the discriminability of a pair of faces; then, all values were linearly scaled between 0 and 1 and metric MDS was applied to approximate a corresponding space. The dimensionality of such spaces was restricted to 20 since that was found sufficient to account for most variance in the data (e.g., over 90% for temporally cumulative analyses). This procedure was conducted on within-expression estimates of discrimination, separately for each expression, resulting in separate spaces for faces with neutral and happy expressions.

Next, we examined face space invariance to image changes introduced by emotional expression. To this end, the fit between neutral and happy face spaces was estimated by aligning one space to the other, via Procrustes transformation, and measuring the badness of fit as the sum of squared errors (SSEs) between the two spaces. Significance testing was then conducted through a permutation test for multidimensional spaces (Jackson, 1995): the labels of each point in one of the two spaces was randomly shuffled and the resulting space was fit to the intact one as above. This procedure was conducted for a total of 10^3^ permutations, by leaving intact each of the two spaces half of the time while permuting the other space, and permutation-based SSE estimates were computed each time.

### Reconstruction approach

The current procedure broadly follows a recently developed approach to facial image reconstruction designed to exploit spatial information in fMRI patterns (Nestor et al., 2016). This procedure capitalizes on the structure of neural-based face space for the purpose of feature derivation and image reconstruction. Here, we deployed this procedure to capture spatiotemporal information in EEG patterns and, further, to examine the ability of expression-invariant visual information to support image reconstructions of facial identity. This procedure was conducted in a sequence of steps as follows.

First, visual features accounting for face space topography were separately derived for each dimension of EEG-based face space. These features were computed as weighted sums of image stimuli following a strategy similar to reverse correlation/image classification (for review, see Murray, 2011; Smith et al., 2012) for applications to EEG data. Hence, here they are referred to as classification images (CIMs). Briefly, face stimuli, following their color conversion to CIEL*a*b*, were summed proportionally to their *z*-scored coordinates on a given dimension of face space. The resulting CIM (i.e., a triplet of images corresponding to L*, a*, and b* channels) amounts to a linear approximation of the visual information responsible for organizing faces along that specific dimension. Thus, for each expression this procedure delivered a total of 20 different CIMs, one for each corresponding dimension of face space.

Second, since not all dimensions may encode systematic visual information, feature/dimension selection was used to identify subspaces relevant for reconstruction purposes. To this end, CIMs corresponding to each dimension were assessed regarding the inclusion of significant information. Specifically, for each dimension, permutation-based CIMs were generated after randomly shuffling the coefficients associated with stimulus images. Then, pixel intensities in the true CIM were compared to the corresponding intensities of pixels in permutation-based CIMs 10^3^ permutations; FDR correction across pixels and color channels) and only CIMs that contained pixel values significantly different from chance were considered for reconstruction purposes.

Third, the coordinates of a target face were estimated in an expression-specific face space. To be clear, the estimation of face space and its CIMs were conducted using all facial identities but one. Then, the left-out face was projected in this space based on its similarity with the other faces. Thus, the procedure ensured that features were not generated from the reconstruction target guarding against circularity.

Last, the target face was constructed through a linear combination of significant CIMs. That is, relevant CIMs, as identified through feature selection above, were summed proportionally with the target’s coordinates in face space and, then, added to an average face obtained from all remaining non-target faces and playing the role of a face prior. However, in contrast to previous work (Nestor et al., 2016), the current procedure capitalized on face space invariance for reconstruction purposes. Specifically, a happy version of face space was aligned to its neutral counterpart via Procrustes transformation using all but one face; then, the left-out face from the happy version of face space was projected into the neutral space using the parameters of the Procrustes mapping function found above. The resulting coordinates in neutral face space were next used to reconstruct the appearance of the target face, with a neutral expression, from neutral CIMs. Conversely, a happy version of the target face relied on aligning neutral face space to its happy counterpart.

Thus, reconstruction relied here on the presence of a robust visual-based structure shared by different face space estimates across expressions. More clearly, in the absence of a common space topography the target face would be projected to a non-informative location of face space and its subsequent reconstruction would resemble the target stimulus no better than expected at chance level (per the evaluation described below).

The procedure above, including face space estimation, was conducted for separate time windows of the ERP trace as well as for temporally cumulative data.

### Evaluation of reconstruction results

Image reconstructions were compared to their target stimuli via two different methods. First, image-based accuracy was estimated as the percentage of instances for which a reconstructed image in CIEL*a*b* was more similar to its target, by a pixel-wise L2 metric, than to any other stimulus with the same expression. Average reconstruction accuracy was then compared against permutation-based chance estimates by shuffling reconstruction labels and by recomputing average accuracy across reconstructions each time (for a total of 10^3^ permutations). This procedure was applied to all types of reconstruction (e.g., both window-based and temporally cumulative) separately for neutral and happy faces.

Second, a single set of reconstructions, based on temporally cumulative group-based data, was subjected to experimental evaluation in a separate behavioral test. To this end, 14 new participants (six males and eight females, age range: 20–28 years) were requested to match image reconstructions to their targets in a two-alternative forced choice (2AFC) task. Specifically, each of 108 unfamiliar face reconstructions, including both expressions, was presented in the company of two stimuli, one of which was the actual target and the other was a foil (another face image). Thus, on each trial, a display was shown containing a reconstructed image, at the top, and two stimuli side by side, at the bottom, all of which had the same expression and the same size (as specified in Experimental procedures). Each display was presented until participants made a response to decide which stimulus was more similar to the top image by pressing a designated left/right key. For each participant, any reconstructed image was presented four times in the company of different foils; thus, across participants, all 53 possible foils for a given reconstruction were exhausted. Stimulus order was pseudorandomized so that different reconstructed images appeared on consecutive trials while target stimuli appeared equally often on the left/right side. Each experimental session was completed over the course of 30 min.

Experimental-based estimates of reconstruction accuracy results were measured as the proportion of correct matches across participants and tested for significance tested against chance (50%) using a one-sample two-tailed *t* test. Last, experimental and homologous image-based estimates of reconstruction accuracy were compared to each other via Pearson’s correlation across images, separately for neutral and happy faces.

## Results

### Univariate ERP results

**Figure 1.**
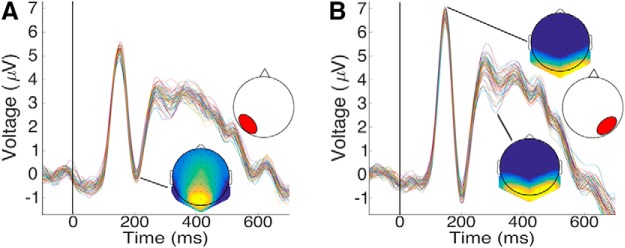
Grand-averaged ERPs across (***A***) left hemisphere electrodes (P5, P7, P9, PO3, PO7, and O1) and (***B***) right hemisphere electrodes (P6, P8, P10, PO4, PO8, and O2) for 54 facial identities (averaged across expressions). Head maps show voltage distributions at (***A***) N170 (***B***) P1, N250.

Three ERP components relevant for face processing, P1, N170, and N250, were each identified across occipitotemporal electrodes (Fig. 1) and examined by a two-way repeated measures ANOVA (54 unfamiliar face identities × two hemispheres). The analysis of the P1 component ^a–c^ (see letter-indexed rows of Table 1 for details of corresponding analyses) and N170^d-f^ analyses found no significant effects. Last, N250 analyses^g-i^ found a main effect of identity (*F*_(53,636)_ = 3.69, *p* = 0.001, *η*_p_^2^ = 0.235) and a marginally significant interaction (*F*_(53,636)_ = 1.32, *p* = 0.07, *η*_p_^2^ = 0.099), both hemispheres^j,k^ showed a significant effect of identity (*F*_(53,636)_ = 2.67, *p* = 0.009, *η*_p_^2^ = 0.182; *F*_(53,636)_ = 3.214, *p* = 0.004, *η*_p_^2^ = 0.21 for the left and right hemispheres, respectively), but the effect was larger on the right side.

**Table 1.** Statistical table

Analysis number	Figure	Description	Data structure	Type of test	Effect	*p* values	Power/CI
a	1	P1 component	Assumed normal	Repeated measures ANOVA	Hemisphere	0.117	0.343
b	1	P1 component	Assumed normal	Repeated measures ANOVA	Identity	0.39	0.447
c	1	P1 component	Assumed normal	Repeated measures ANOVA	Identity X hemisphere	0.551	0.311
d	1	N170 component	Assumed normal	Repeated measures ANOVA	Hemisphere	0.146	0.299
e	1	N170 component	Assumed normal	Repeated measures ANOVA	Identity	0.513	0.373
f	1	N170 component	Assumed normal	Repeated measures ANOVA	Identity X hemisphere	0.307	0.532
g	1	N250 component	Assumed normal	Repeated measures ANOVA	Hemisphere	0.171	0.269
h	1	N250 component	Assumed normal	Repeated measures ANOVA	Identity	0.001	0.980
i	1	N250 component	Assumed normal	Repeated measures ANOVA	Identity X hemisphere	0.07	0.560
j	1	N250 component	Assumed normal	Repeated measures ANOVA	Identity in LH	0.009	0.926
k	1	N250 component	Assumed normal	Repeated measures ANOVA	Identity in RH	0.004	0.943
l	2*A*	Group-based discrimination	Normality not assumed	Permutation test	Across expression	FDR-corrected *p* = 0.006	
m	2*B*	Representative participant discrimination	Normality not assumed	Permutation test	Across-expression	FDR-corrected *p* = 0.004	
n	3*A*	Group-based discrimination	Normality not assumed	Permutation test	Across expression/neutral	0.001	95% CI: 47.1–51.3
o	3*A*	Group-based discrimination	Normality not assumed	Permutation test	Across expression/happy	0.001	95% CI: 47.2–50.9
p	3*A*	Group-based discrimination	Normality not assumed	Permutation test	Within expression/neutral	0.001	95% CI: 45.9–51.8
q	3*A*	Group-based discrimination	Normality not assumed	Permutation test	Within expression/happy	0.001	95% CI: 45.9–52.1
r	3*B*	Single-participant-based discrimination	Assumed normal	Two-tailed *t* test	Across expression/neutral	0.001	95% CI: 53.1–57
s	3*B*	Single-participant-based discrimination	Assumed normal	Two-tailed *t* test	Across expression/happy	0.001	95% CI: 53.2–57.2
t	3*B*	Single-participant-based discrimination	Assumed normal	Two-tailed *t* test	Within expression/neutral	0.001	95% CI: 0.559–0.602
u	3*B*	Single-participant-based discrimination	Assumed normal	Two-tailed *t* test	Within expression/`happy	0.001	95% CI: 54.8–60.4
v	n/a	Single-participant-based discrimination	Assumed normal	Repeated measures ANOVA	Discrimination type	<0.001	>0.999
w	n/a	Single-participant-based discrimination	Assumed normal	Repeated measures ANOVA	Expression	0.466	0.107
x	n/a	Single-participant-based discrimination	Assumed normal	Repeated measures ANOVA	Discrimination type X expression	0.211	0.230
y	n/a	Single-participant-based discrimination within and across databases	Assumed normal	Repeated measures ANOVA	Discrimination type	<0.001	>0.999
z	n/a	Single-participant-based discrimination within and across databases	Assumed normal	Repeated measures ANOVA	Pairs type	<0.001	>0.999
aa	n/a	Single-participant-based discrimination within and across databases	Assumed normal	Repeated measures ANOVA	Discrimination type X pairs type	0.033	0.565
ab	n/a	Single-participant-based discrimination within and across databases	Assumed normal	*Post hoc* (matched two-tailed *t* test)	Across-within database for within-expression versus across-within database for across-expression	0.412	95% CI: –0.1–1.5
ac	n/a	Single-participant-based discrimination within and across databases	Assumed normal	Two-tailed *t* test against 0.5	Within-expression, within-database discrimination	<0.001	95% CI: 54.6– 57.9
ad	n/a	Single-participant-based discrimination within and across databases	Assumed normal	Two-tailed *t* test against 0.5	Within-expression, across-database discrimination	<0.001	95% CI: 56.4–61
ae	n/a	Single-participant-based discrimination within and across databases	Assumed normal	Two-tailed *t* test against 0.5	Across-expression, within-database discrimination	<0.001	95% CI: 52.8–55
af	n/a	Single-participant-based discrimination within and across databases	Assumed normal	Two-tailed *t* test against 0.5	Across-expression, across-database discrimination	<0.001	95% CI: 54–57.7
ag	n/a	temporally cumulative analysis	Assumed normal	Pearson’s correlation	EEG-based discrimination/behavioral discrimination	<0.001	0.436

ah	4	Temporal correlation	Assumed normal	Pearson’s correlation	Across-expression discrimination/behavioral discrimination	FDR-corrected *p* = 0.002	
ai	n/a	Temporally cumulative analysis	Assumed normal	Pearson’s correlation	Across-expression discrimination/CFMT	0.219	0.237
aj	n/a	Temporally cumulative analysis	Assumed normal	Pearson’s correlation	Across-expression discrimination/VVIQ-2	0.676	0.05
ak	5*B*	Face space fit	Normality not assumed	Permutation test	Happy-neutral similarity	<0.001	95% CI: 0.738–0.798
al	6*A*	CIM	Normality not assumed	Permutation test	Neutral dimension 1; luminance	FDR-corrected *p* = 0.027	
am	6*A*	CIM	Normality not assumed	Permutation test	Neutral dimension 1; red-green	FDR-corrected *p* = 0.018	
an	6*A*	CIM	Normality not assumed	Permutation test	Neutral dimension 1; yellow-blue	FDR-corrected *p* = 0.024	
ao	6*A*	CIM	Normality not assumed	Permutation test	Neutral dimension 2; luminance	FDR-corrected *p* = 0.012	
ap	6*A*	CIM	Normality not assumed	Permutation test	Neutral dimension 2; red-green	N/A	
aq	6*A*	CIM	Normality not assumed	Permutation test	Neutral dimension 2; yellow-blue	FDR-corrected *p* = 0.009	
ar	6*B*	CIM	Normality not assumed	Permutation test	Happy dimension 1; luminance	FDR-corrected *p* = 0.040	
as	6*B*	CIM	Normality not assumed	Permutation test	Happy dimension 1; red-green	FDR-corrected *p* = 0.018	
at	6*B*	CIM	Normality not assumed	Permutation test	Happy dimension 1; yellow-blue	FDR-corrected *p* = 0.024	
au	6*B*	CIM	Normality not assumed	Permutation test	Happy dimension 2; luminance	N/A	
av	6*B*	CIM	Normality not assumed	Permutation test	Happy dimension 2; red-green	FDR-corrected *p* = 0.021	
aw	6*B*	CIM	Normality not assumed	Permutation test	Happy dimension 2; yellow-blue	FDR-corrected *p* = 0.008	
ax	7*B*	Temporal reconstruction accuracy	Normality not assumed	Permutation test	Neutral	FDR-corrected *p* = 0.002	
ay	7*B*	Temporal reconstruction accuracy	Normality not assumed	Permutation test	Happy	FDR-corrected *p* = 0.006	
bz	8*B*	Reconstruction accuracy (image-based)	Normality not assumed	Permutation test	Neutral	0.001	95% CI: 42.3–57.8
ba	8*B*	Reconstruction accuracy (image-based)	Normality not assumed	Permutation test	Happy	0.001	95% CI: 42.1–58.1
bb	8*C*	Reconstruction accuracy (experimental-based)	Assumed normal	Two-tailed *t* test	Neutral	0.001	95% CI: 55.6–62.6
bc	8*C*	Reconstruction accuracy (experimental-based)	Assumed normal	Two-tailed *t* test	Happy	0.001	95% CI: 52.4–59.2
bd	n/a	Correlation between experimental and image-based accuracies	Assumed normal	Pearson’s correlation	Neutral	0.001	0.912
be	n/a	Correlation between experimental and image-based accuracies	Assumed normal	Pearson’s correlation	Happy	0.002	0.898
bf	n/a	Correlation between reconstruction and discrimination	Assumed normal	Pearson’s correlation	Averaged across expressions	<0.001	>0.999
bg	n/a	Single-participant-based reconstruction accuracy (image-based)	Assumed normal	Two-tailed *t* test	Neutral	0.027	0.676
bh	n/a	Single-participant-based reconstruction accuracy (image-based)	Assumed normal	Two-tailed *t* test	Happy	0.045	0.580
bi	n/a	Correlation between single-participant-based reconstruction and discrimination	Assumed normal	Pearson’s correlation	Neutral	<0.001	0.974
bj	n/a	Correlation between single-participant-based reconstruction and discrimination	Assumed normal	Pearson’s correlation	Happy	<0.001	0.980

### Pattern classification of facial identity

A total of 108 unfamiliar male faces (54 individuals × two emotional expressions) were classified based on ERP traces associated with their viewing. Specifically, spatiotemporal ERP patterns across bilateral occipitotemporal electrodes were averaged across participants and, then, evaluated for their ability to support facial identity discrimination. To assess the time course of individual face processing, classification was conducted across consecutive 10-ms temporal windows both within and across expression by training and testing the classifier on faces with the same or different expression.

**Figure 2.**
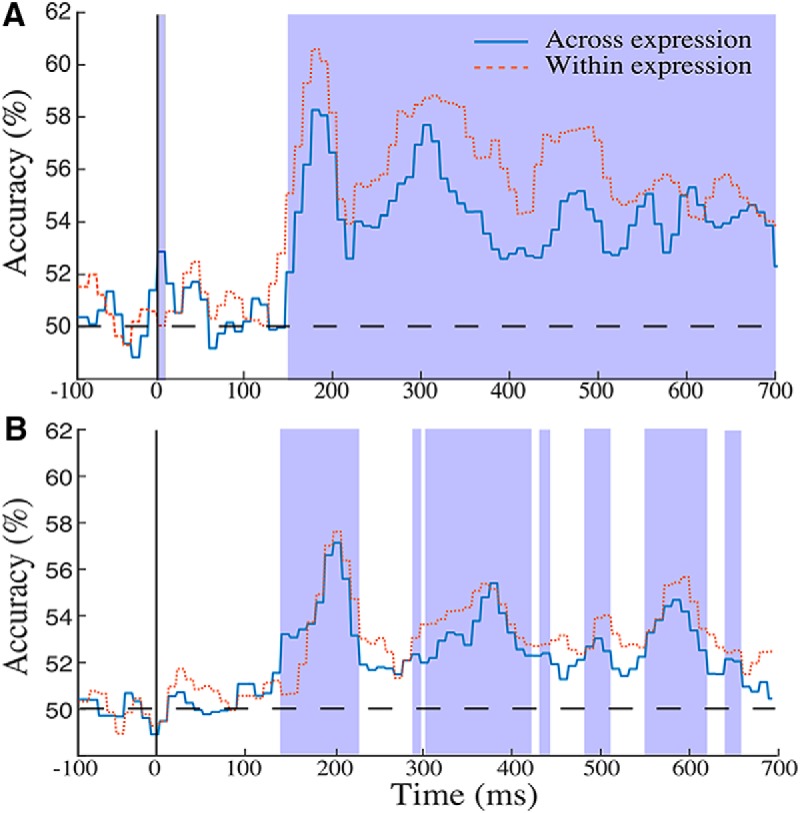
The time course of EEG-based classification accuracy for across- and within-expression discrimination of facial identity. ***A***, Classification was conducted across consecutive 10-ms window patterns over 12 occipitotemporal electrodes for group-based ERP data. Both types of analysis exhibited above-chance discrimination across extensive temporal intervals (permutation test; FDR correction across time, q < 0.01); shaded areas mark intervals of better-than-chance discrimination for across-expression classification. ***B***, The time course of EEG-based classification accuracy for across- and within-expression discrimination of facial identity for a single representative participant. Classification was conducted across consecutive 10-ms window patterns over 12 occipitotemporal electrodes. Both types of analysis exhibited above-chance discrimination across extensive temporal intervals (permutation test; FDR correction across time, q < 0.01); shaded areas mark intervals of better-than-chance discrimination for across-expression classification.

This analysis found significant levels of discrimination across extensive intervals^l^ (permutation test; *q* < 0.01). Specifically, across-expression classification evinced above-chance accuracy from 152 ms after stimulus presentation until the end of the epoch, with two peaks at 170 and 295 ms (see Fig. 2*A* for the group-based results). Within-expression classification yielded a similar time course but consistently higher levels of accuracy and an earlier onset, at 140 ms, in agreement with the reliance on additional, lower-level image cues for this type of discrimination (In addition, an earlier interval of significance was found for across-expression classification between 0-5 ms; however, given its very early occurrence, its reduced amplitude and the absence of its replication by within-expression classification we treat this data point as a false positive). Of note, for both types of classification we found that discrimination accuracy was maximized in the vicinity of the N170 component as identified by univariate analyses of ERP data (Fig. 1).

Following the approach described above group-based analyses were complemented next by single-participant analyses^m^. These analyses confirmed the feasibility of facial identity discrimination from the data of single participants (see Fig. 2*B* for the results of a representative participant). However, discrimination levels were lower than in the group-based analyses, likely due to the lower SNR of single-participant ERPs and its impact on classification (Grootswagers et al., 2017). Further, multiple intervals of discrimination emerged, as opposed to a single, uninterrupted one.

Next, pattern classification was applied to temporally cumulative data by concatenating data points from all time bins between 50 and 650 ms after stimulus onset^n-q^. The aim of this analysis was to maximize discrimination performance by concomitantly exploiting relevant information from all potentially relevant time points. Specifically, while the ∼61-fold increase in pattern dimensionality (i.e., 12 electrodes × 307 time bins) would, by itself, reduce the effectiveness of classification, we considered the possibility that any ensuing classification decrement may be offset by the use of complementary sources of information from different time points (Blankertz et al., 2011).

**Figure 3.**
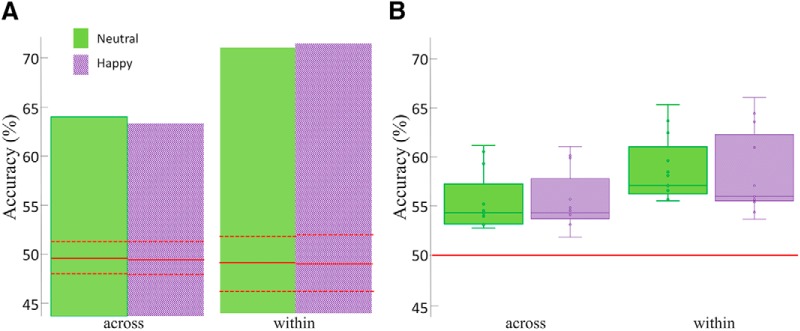
EEG-based classification accuracy for across- and within-expression discrimination of facial identity with temporally cumulative data (50–650 ms after stimulus onset). Accuracy corresponding to neutral and happy faces are separately shown for (***A***) group-based ERP data and (***B***) single-participant data (i.e., pattern classification was conducted individually for each participant and, then, its results averaged across participants). The plots display (***A***) the results of permutation tests (red solid and dash lines indicate average accuracy and 99% confidence intervals estimated with 10^3^ permutations) and (***B***) the distribution of single-participant data (green and purple solid lines indicate medians, boxes represent 1st and 3rd quartiles, whiskers represent minimum and maximum accuracy values, points represent individual participants’ values and red solid lines indicate chance-level discrimination).

Consistent with the hypothesis above, we found that this analysis yielded robust levels of discrimination for group-based data (Fig. 3*A*): 64% and 71% for across- and within-expression discrimination, respectively (permutation test; *p* = 0.001 for both types of discrimination and both expressions). Of note, these results outperform peak performance obtained with window-based analyses in the proximity of the N170 component. Further, single-participant estimates of discrimination with temporally cumulative data were also computed^r-u^ and, then, averaged across participants (Fig. 3*B*). Again, performance was better than chance for both within-expression discrimination (two-tailed *t test* across participants against 50% chance-level discrimination; *t*_(12)_ = 9.89 and 7.27, Cohen’s *d* = 2.1 and 2.86 for neutral and happy faces, respectively; *p* values = 0.001) and for across-expression discrimination (*t*_(12)_ = 6.84 and 7; *d* = 2.02 and 1.97 for neutral and happy faces, respectively; *p* values < 0.001). Further, a two-way repeated measures ANOVA^v-x^ (two discrimination types × two expressions) revealed a main effect of discrimination types (*F*_(1,12)_ = 50.05, *p* < 0.001, η_p_^2^ = 0.81), with higher accuracy for within than across-expression discrimination, but no effect of expression and no interaction.

To examine possible effects of face database the analysis above was repeated while restricting pattern classification either to pairs of faces from the same database or from different databases. A two-way repeated measures ANOVA^y-ab^ (two discrimination types: within/across expression × two pair types: within/across database) was conducted to this end – classification estimates were collapsed across neutral and happy faces given the absence of any expression effects above. This analysis revealed a main effect of discrimination types (*F*_(1,12)_ = 45.92, *p* < 0.001, η_p_^2^ = 0.79), with higher accuracy for within than across-expression discrimination, as well as a main effect of pair types (*F*_(1,12)_ = 38.73, *p* < 0.001, η_p_^2^ = 0.76), with higher accuracy for within than across-database classification. Critically though, all classification estimates were significantly above chance (two-tailed *t* tests against 50% chance-level discrimination)^ac-af^; mean accuracy = 56.3%, *t*_(12)_ = 8.36, *p* < 0.001, Cohen’s *d* = 2.32 for within-expression, within-database discrimination; mean accuracy = 58.7%, *t*_(12)_ = 8.38, *p* < 0.001, Cohen’s *d* = 2.32 for within-expression, across-database discrimination; mean accuracy = 53.9%, *t*_(12)_ = 7.64, *p* < 0.001, Cohen’s *d* = 2.12 for across-expression, within-database discrimination**;** mean accuracy = 55.9%, *t*_(12)_ = 6.88, *p* < 0.001, Cohen’s *d* = 1.91 for across-expression, across-database discrimination).

Last, for completeness, classification analyses including all face pairs within and across databases were repeated with all 64 electrodes, instead of the subset of 12 occipitotemporal electrodes noted above. However, no consistent boost in discrimination was found for any analysis in this case. Hence, for simplicity, the remainder of our analyses and results, as reported below, are based on occipitotemporal electrodes only.

### Neural-based discrimination and behavioral performance

**Figure 4.**
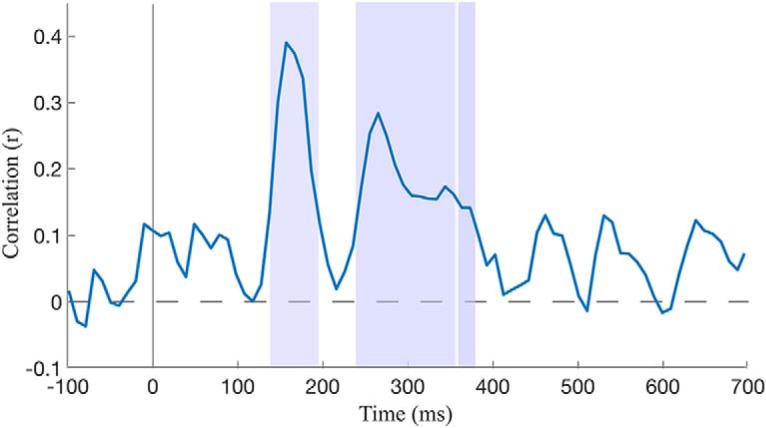
Correlation of EEG- and behavioral-based estimates of pairwise face similarity. EEG-based estimates are derived from across-expression discrimination of facial identity for consecutive 10-ms windows of group-based data. Multiple intervals, marked by shaded areas, exhibit significant levels of correlation (permutation test; FDR correction across time, q < 0.01).

To assess and to quantify the relationship between behavioral and neural-based face similarity, group-level estimates of EEG-based discrimination were related to behavioral estimates of pairwise face similarity. Specifically, across-expression discrimination accuracy was compared with its behavioral counterpart, across identity pairs, through Pearson’s correlation^ag^. This comparison revealed, first, a significant correlation for discrimination estimates from the temporally cumulative analysis (*r* = 0.245; *p* < 0.001). Then, behavioral estimates were correlated with their neural counterpart separately for each 10-ms temporal window (Fig. 4)^ah^. This analysis found multiple intervals of significant correlation, between 137 and 197, 236 and 335, and 340 and 385 ms, with peaks at 157 and 266 ms (*q* < 0.01).

Next, we sought to assess whether the different levels of discrimination achieved with different participants were related to their overall face processing skills. To this end, individual estimates of discrimination from the temporally cumulative analysis, averaged across identity pairs, were correlated with CFMT^ai^ scores of the same individuals. No significant correlations were found with these scores or with any other behavioral measures considered such as average familiarity ratings with famous faces or VVIQ-2^aj^ scores.

### Neural-based face space and expression-invariance

Face space estimates were derived through the application of MDS to within-expression face classification of temporally cumulative data. Specifically, MDS was applied to pairwise face discrimination values derived through pattern analysis of group-based data, separately for neutral and happy faces. Then, the resulting spaces were reduced to the first 20 dimensions and aligned with each other via Procrustes transformation.

**Figure 5.**
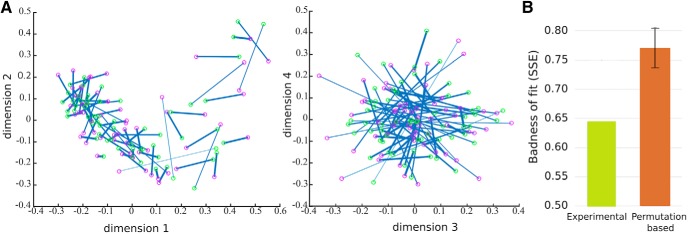
Neutral and happy face space estimates along with their fit (after Procrustes alignment). Estimates were derived through MDS analysis of similarity matrices based on within-expression face discrimination of group-based temporally cumulative data. The two face space estimates exhibit a similar topography as found with (***A***) their visualization across multiple dimensions (red and green circles indicate neutral and happy faces, respectively; solid lines connect face images with the same identity with the thickness of the line proportionally reflecting shorter distances; the first four dimensions shown here account for 40% and 41% variance for neutral and happy face space); (***B***) badness of fit (SSEs) for the two spaces compared to their permutation-based counterpart (average fits and 95% confidence intervals estimated with 10^3^ permutations).

An examination of the resulting spaces for neutral and happy faces, following their alignment, seemed consistent with the presence of a common topography across expressions (Fig. 5*A*). To assess their fit more rigorously a comparison with permutation-based alignment estimates (Fig. 5*B*)^ak^ was computed next. This comparison indicated that the fit between the two spaces was considerably better than chance (*p* < 0.001). Beyond its theoretical implications, this finding is relevant here in that it may allow exploiting the structure of visual information invariant across expression for reconstruction purposes, as detailed next.

### Reconstruction results

Visual features were derived from the structure of face space, dimension by dimension, through a procedure akin to reverse correlation/image classification (Gosselin and Schyns, 2003; Sekuler et al., 2004; Martin-Malivel et al., 2006). Such features, or CIMs, were then assessed through a permutation test for the presence of significant information pixel by pixel separately for each CIEL*a*b* color channel.

**Figure 6.**
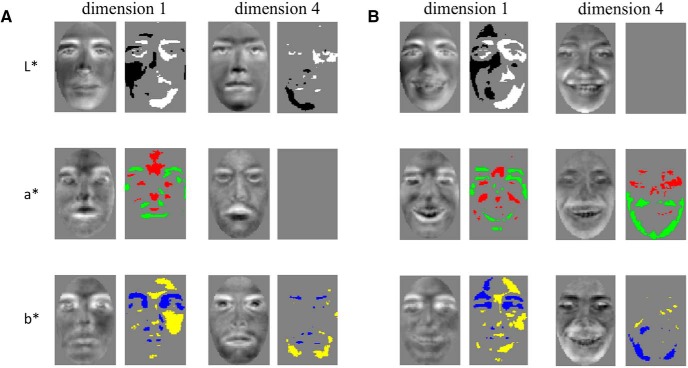
Examples of CIMs extracted from EEG-based face space constructs for (***A***) neutral and (***B***) happy faces. Pairs of images show raw CIMs (odd columns) and their analysis (even columns) with a pixelwise permutation-based test (FDR-corrected across pixels; q < 0.05). Bright/dark, red/green, and yellow/blue regions in analyzed CIs mark areas of the face brighter (L*), redder (a*), or more yellow (b*) than chance in CIEL*a*b*. Results are shown separately for the first and fourth dimensions of face spaces derived from group-based temporally cumulative data.

An examination of CIMs containing significant^al-aw^ information revealed global contrast patterns across multiple color channels, examples of such features derived from group-based temporally cumulative data are shown in Figure 6. The emergence of these features confirmed that neural-based face space is, at least partly, organized by visual information (as opposed, for instance, to higher-level semantic information). More relevantly here, it points to the potential value of CIMs as reconstruction features.

Accordingly, significant CIMs were linearly combined to deliver an approximation of face appearance broadly following an image reconstruction approach recently used with fMRI data (Nestor et al., 2016). Specifically, image reconstruction was separately applied to neutral and happy expressions. As noted above, the common face space topography for the two expressions could, in theory, allow using the relative position of a given identity in one space to deliver a reconstruction of the same facial identity with the opposite expression.

**Figure 7.**
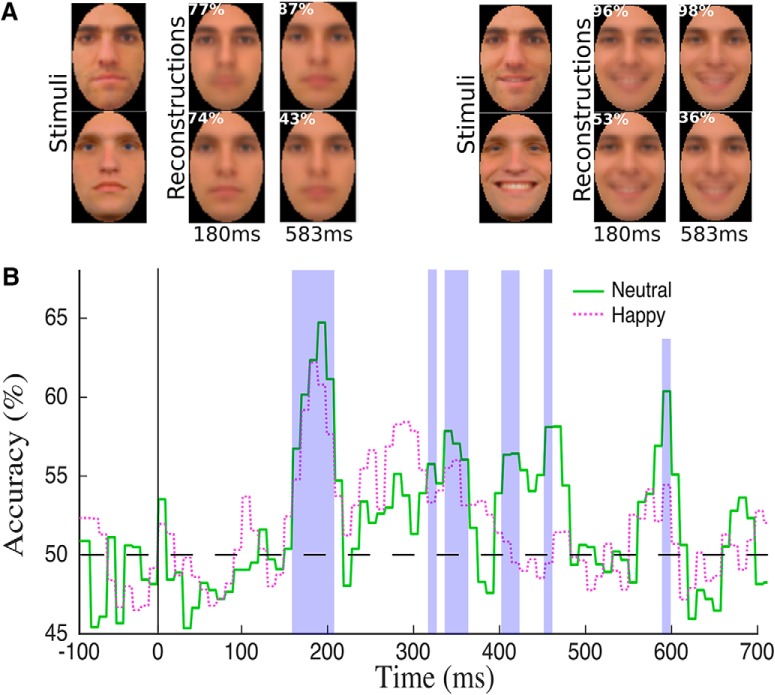
Reconstruction results for neutral and happy face images across consecutive 10-ms windows of group-based data. ***A***, Examples of face stimuli along with their corresponding reconstructions at two different times (numbers in the upper left corner indicate image-based estimates of reconstruction accuracy). ***B***, Time course of reconstruction accuracy. Both neutral and happy face images exhibit above-chance discrimination across multiple temporal intervals (permutation test; FDR correction across time, q < 0.05; shaded areas mark intervals of better-than-chance discrimination for neutral faces). Reconstruction accuracy is maximized in the vicinity of the N170 component (Fig. 1) and of the discrimination peak found with pattern classification (Fig. 2).

Consistent with the hypothesis above, this procedure, as performed for separate time windows with group-based data, found evidence for multiple intervals capable of supporting above-chance reconstruction (image-based permutation test; *q* < 0.05), reconstruction accuracy was assessed via a pixelwise image-matching test across reconstructed images and stimulus images. The earliest interval had an onset at 160 and 170 ms for neutral^ax^ and happy ^ay^ faces, respectively, while accuracy peaked at 187 and 180 ms for the two expressions. Examples of reconstructions, converted from CIEL*a*b* back to RGB, are shown in Figure 7*A* for two different time points while average reconstruction accuracies are plotted in Figure 7*B*. Further, a representative example of image reconstruction for a single face, interval by interval, is shown in Movie 1 along with the temporal profile of its reconstruction accuracy.

Movie 1. Illustration of (top) neutral face stimulus and its reconstruction across 10-ms windows of group-based data along with (bottom) the time course of its reconstruction accuracy assessed with an image-based test. The last frame of the movie shows (top) facial image reconstruction achieved with temporally cumulative data and (***B***) its corresponding level of accuracy (dashed red line). 10.1523/ENEURO.0358-17.2018.video.1

**Figure 8.**
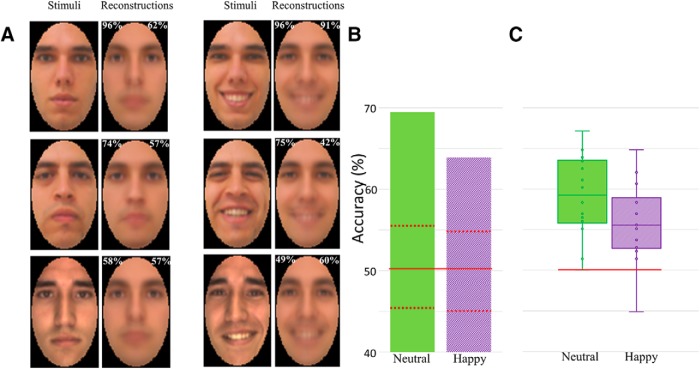
Reconstruction results for neutral and happy faces relying on temporally cumulative group-based data. (***A***) Examples of face stimuli along with their corresponding reconstructions (numbers in the upper left corner indicate image-based estimates of reconstruction accuracy; numbers in the upper right indicate experimental-based accuracy). ***B***, Average image-based reconstruction accuracy (red solid and dash lines indicate average accuracy and 95% confidence intervals estimated with 10^3^ permutations). ***C***, Average experimental-based reconstruction accuracy (green and purple solid lines indicate medians, boxes represent 1st and 3rd quartiles, whiskers represent minimum and maximum accuracy values, points represent individual participants’ values and red solid lines indicate chance-level reconstruction).

The application of the same procedure to temporally cumulative data led to more robust results: 69.46% image-based accuracy for neutral faces^az^ and 63.91% for happy faces^ba^ (permutation test; *p* values = 0.001). Figure 8*A* shows examples of reconstructions and Figure 8*B* displays average accuracy estimates and their permutation-based assessment. Experimental-based estimates of accuracy, obtained with a new group of participants, led to more modest levels of accuracy (Fig. 8*C*); however, both neutral and happy face reconstructions were still accurate above chance (two-tailed *t* test across participants against 50% chance-level discrimination: *t*_(13)_ = 6.70, 4.38; Cohen’s *d* = 1.86, 1.22, for neutral^bb^ and happy^bc^, respectively; *p* < 0.001 for all), with no difference between neutral and happy faces. Further, reconstruction accuracies as estimated by the two tests, image-based and experimental-based, were compared with each other across facial identities and were found to significantly correlate with each other (*r* = 0.43 and 0.42; *p* = 0.001 and 0.002 for neutral^bd^ and happy^be^ faces, respectively), thus mutually reinforcing their validity.

Next, across-expression classification estimates obtained with temporally cumulative data were compared with their corresponding reconstruction accuracies averaged across expressions. Specifically, Pearson’s correlation across facial identities found a positive relationship between across-expression discrimination and image-based accuracy^bf^ (*r* = 0.87, *p* < 0.001). Thus, the more discriminable a facial identity is, the more accurately it can be reconstructed.

Last, reconstruction was performed for single-participant data and evaluated with the aid of the image-based test. Accuracy levels were still above chance (two-tailed *t* test across participants against 50% chance-level discrimination; mean accuracy = 53.1%, *t*_(12)_ = 2.52, *p* = 0.027, Cohen’s *d* = 0.73 and mean accuracy = 53.3%, *t*_(12)_ = 2.24, *p* = 0.045, Cohen’s *d* = 0.65 for neutral^bg^ and happy^bh^ face reconstructions, respectively) while Pearson’s correlations between classification accuracy and reconstruction accuracy across participants were also found significant (*r* = 0.83 and *r* = 0.84, for neutral^bi^ and happy^bj^ faces, respectively; *p* values < 0.001). Thus, participants who provided data supporting higher levels of face classification also provided more accurate reconstruction results.

## Discussion

The current work investigates the neural basis of individual face processing and its temporal dynamics through the application of pattern analysis and image reconstruction to EEG data. This investigation yields several notable outcomes as follows.

First, we find that EEG data support facial identity discrimination. By and large, this finding confirms the possibility of EEG-based pattern classification of facial identity across changes in expression from EEG data (Nemrodov et al., 2016). Discrimination peaks were identified in the proximity of the N170 and N250 ERP components, consistent with univariate analyses pointing to the relevance of the former (Itier and Taylor, 2002; Heisz et al., 2006; Jacques et al., 2007; Caharel et al., 2009) and the latter (Schweinberger et al., 2002; Huddy et al., 2003; Tanaka et al., 2006) for face processing. The onset of discrimination, around 150 ms, was intermediary to early estimates in the vicinity of P1 (Nemrodov et al., 2016) and later estimates around 200 ms as reported with MEG (Vida et al., 2017). One possibility is that early estimates, along with higher levels of discrimination, can be triggered by the use of low-level image properties (Cauchoix et al., 2014; Ghuman et al., 2014). In line with this consideration, we found that within versus across-expression discrimination produced earlier and consistently higher levels of discrimination accuracy. Importantly though, across-expression classification, which aims to minimize reliance on low-level cues, exhibited robust levels of discrimination across an extensive interval (i.e., from ∼150 ms onwards) while its time course was also mirrored by that of neural-behavioral correlations in the context of pairwise face similarity.

Second, temporally cumulative analyses targeted identity discrimination across a broad interval between 50 and 650 ms after stimulus onset. Despite the increase in dimensionality for the classification patterns, these data supported even more robust levels of accuracy for both within and across-expression discrimination, consistent with the presence of relevant information at multiple time points. Moreover, the superior levels of discrimination obtained with temporally cumulative data, as opposed to 10-ms windows, agrees with the presence of distinct sources of information at different time points. That is, we relate the boost in classification accuracy with the ability to exploit complementary information about facial identity at different times. Interestingly, this conclusion echoes that based on the lack of temporal generalization found with cross-temporal object decoding of MEG data (Carlson et al., 2013; Isik et al., 2014), specifically, the lack of classification success with using training and testing data from distinct time intervals has been taken as evidence for the presence of different types of information over time (Grootswagers et al., 2017). Further, the boost in classification noted above is important for practical purposes: it suggests that investigations that place less emphasis on clarifying the time course of discrimination can be better served by exploiting patterns across larger temporal intervals. Accordingly, our subsequent investigations into face space structure and image reconstruction were conducted with both window-based and cumulative data.

Third, a neural-based estimate of face space was constructed from EEG data and its organization was explained by the presence of visual information captured by CIMs. This result is significant in that it confirms that pattern discrimination relies, at least partly, on relevant visual information (e.g., as opposed to higher-level semantic cues). More importantly, we note that neural-based face space has been examined in the context of fMRI (Loffler et al., 2005; Rotshtein et al., 2005; Gao and Wilson, 2013) and monkey neurophysiology (Leopold et al., 2006; Freiwald et al., 2009). Yet, many of its properties, as related to facial identity representation, remain to be clarified. For instance, its invariant structure across different types of image transformation remains to be assessed and quantified. Behavioral research suggests that face space topography is largely invariant across viewpoint and lighting (Blank and Yovel, 2011). Here, we reach a similar conclusion regarding the expression invariance of neural-based face space as derived from EEG data.

Fourth, image reconstruction was conducted with the aid of CIM features derived directly from the structure of EEG data (i.e., as opposed to predefined visual features selected due to their general biological plausibility). This endeavor builds on pattern classification while, critically, it validates its results by showcasing its reliance on relevant visual information encoded in the EEG signal. We found that multiple temporal intervals supported better-than-chance reconstruction for both neutral and happy faces with a peak in the proximity of the N170 component. Also, reconstruction accuracy was further boosted by considering temporally cumulative information, as used for pattern classification. More importantly, these results are notable in that, unlike previous work with fMRI-based facial image reconstruction (Cowen et al., 2014; Nestor et al., 2016), they exploit invariant face space information for reconstruction purposes. Thus, arguably the current findings speak to the visual nature of facial identity representations rather than just to lower-level pictorial aspects of face perception.

Further, the current work provides proof of principle for EEG-based image reconstruction. Importantly, not only does this demonstrate the applicability of image reconstruction to neuroimaging modalities other than fMRI but, critically, it shows that EEG-based reconstruction can compete in terms of overall accuracy with its fMRI counterpart (Nestor et al., 2016).

Thus, here we build on previous EEG investigations of face processing and on pattern analyses of neuroimaging data to address several theoretical and methodological issues. In particular, the current work capitalizes on previous attempts at clarifying the temporal profile of individual face processing via linear classification of spatiotemporal EEG patterns across facial expression (Nemrodov et al., 2016). In agreement with this previous work we find that individual faces can be discriminated from their corresponding EEG patterns, that their time course exhibits an extended interval of significant discrimination and that multiple discrimination peaks occur, including an early one in the vicinity of the N170 component. Unlike this previous work though, which only relied on a restricted set of eight male and female faces, we find that such discrimination can be performed even with a large, homogenous set of face images controlled for low and high-level face properties (e.g., through geometrical alignment and intensity normalization of 108 white male face images). Hence, differences in discrimination onset across studies (i.e., 70 ms in this previous work vs 152 ms here) are likely related to reliance on idiosyncratic image differences within a small stimulus set in this previous investigation. More importantly though, not only does the current work examine the time course of individual face classification in a more reliable and thorough manner but, critically, it utilizes its outcomes for the purpose of facial feature derivation and image reconstruction.

Naturally, boosting even further classification and reconstruction accuracy is an important future endeavor. Regarding classification, this could be achieved, for instance, through efficient techniques for feature selection, such as recursive feature elimination (Hanson and Halchenko, 2008; Nestor et al., 2011), aimed at reducing pattern dimensionality and optimizing discrimination performance. Since electrodes are likely to carry irrelevant or redundant information at multiple time points, eliminating this information from higher-dimensional spatiotemporal patterns (e.g., across all electrodes and time points) could benefit classification. Regarding reconstruction, more complex, biologically-plausible approaches can be developed, for instance, by considering shape and surface information separately within the reconstruction process. Since shape and surface provide complementary cues to face processing (Jiang et al., 2006; Andrews et al., 2016), it would be informative to derive separate types of CIMs corresponding to this distinction and to consider their separate contribution to facial image reconstruction.

Notably, however, beyond overall performance, EEG-based reconstruction stands out by its ability to clarify the dynamics of visual representations as they develop in response to a given stimulus. For instance, it can speak to how a percept evolves over time in response to a static stimulus, as attempted here, by inspecting image reconstruction across consecutive time windows. Alternatively, this method could be extended to recover fine-grained dynamic information as present in moving stimuli. While reconstruction of natural movies has been previously conducted with fMRI (Nishimoto et al., 2011), the superior temporal resolution of EEG could make this modality a more efficient choice for the recovery of dynamic visual information. Further, we note that the comparatively wide availability of EEG systems could also render this modality the preferred choice for the development of new types of image-reconstruction brain-computer interfaces.

Last, while the current investigation focuses on faces as a visual category of interest, we argue that the present methodological approach can inform individual object recognition more generally. This is theoretically suggested by the presence of common neurocomputational principles underlying face and object identification (Cowell and Cottrell, 2013; Wang et al., 2016) as well as, methodologically, by the ability to evaluate the dynamics of invariant object recognition (Isik, et al., 2014). Particularly encouraging in this sense is the success of efforts to construct and characterize object similarity spaces from MEG (Carlson et al., 2013) and EEG data (Kaneshiro et al., 2015).

To conclude, our investigation targets the neural dynamics of face processing as reflected by EEG patterns. Our findings shed new light on the time course of facial identity processing while providing a way to extract and to assess the underlying visual information. Last, from a methodological standpoint, our results establish the feasibility of EEG-based image reconstruction and, more generally, they confirm the rich informational content of spatiotemporal EEG patterns.

## Synthesis

Reviewing Editor: Bradley Postle, University of Wisconsin Decisions are customarily a result of the Reviewing Editor and the peer reviewers coming together and discussing their recommendations until a consensus is reached. When revisions are invited, a fact-based synthesis statement explaining their decision and outlining what is needed to prepare a revision will be listed below. The following reviewer(s) agreed to reveal their identity: Emmanuel Barbeau

Here are the two reviewers' comments, in full:

Reviewer #1

The authors expand on previous (presumably their) work by demonstrating that the information contained in EEG signals is not only sufficient for face identity classification but also robust enough for face reconstruction. Overall the authors did an excellent work and I only have minor comments:  

From Figures 2A, 7B and the supplemental movie it appears that classification/reconstruction spikes momentarily at around 0 (stimulus onset): at time t=-25ms (before stimulus onset), reconstruction accuracy is at 69% in the supplemental movie the authors provided. At the group level this is also evident but less so (at t=0 accuracy is around 54%).This is surprising and raises the question of what information the classification algorithm is picking up at that early stage. I understand that the authors controlled the luminance and RMS contrast of their stimuli but I am wondering if the classifiers are still picking up on lower-level differences between the faces. Perhaps the authors can address this comment in the discussion section.

Reviewer #2

In this study the authors use a combination of decoding and image reconstruction techniques to explore how EEG recorded from the scalp is related to face identity. The most interesting part of this study is the demonstration that it is possible to classify and reconstruct individual faces from EEG with methodological approaches that are well described. With some exceptions, most previous reported had focused on category decoding rather than on individual faces, which makes this study an interesting contribution. In contrast, the contribution of this study to the understanding of the time course of face processing is more minor. 

I have a few questions/suggestions: 

- the authors use in their title the term “facial identity processing” which is appropriate. However they also sometimes use in the article (and abstract) the term “face recognition” which is rather vague, and in fact confusing because there is no relation in this study with familiarity or semantic processing. 

- Have the authors included all subjects that were recorded (13) or have they excluded some? If yes, explain why. 

- Have the authors verified if there was an effect of the database from which the faces were chosen? 

- In the Methods, it is sometimes difficult to follow the exact number of trials/face that went into each analysis. The authors start from 64 trials/faces (this is not specifically written), then average trials to end up with 16 EEG trials/faces (which is something we understand two pages later). As this is critical for any future work that will aim at reducing the number of trials necessary for suitable decoding, all this could be more explicit or discussed. 

- A related question is why the authors chose to average EEG trials by group of 4. Did they make specific test about this? 

- The results about the accuracy levels for single-participants reconstructions are not provided (only the p values are). Please correct. I suppose the “d” mentioned in this section (p 22, line 45) refer to Cohen's d. Please clarify. 

- The Nemrodov et al (2016) study is related to the present one. It is briefly mentioned in the Discussion but should be discussed more extensively, both for its results and methodology. 

- Please change all histograms to boxplots and distribution of individual results. Those are much more informative. 

Typos and other: 

- I couldn't find the Naselaris in the references (p3, line 39). 

- p11, line 28, correct ‘data. and’. 

- p22, line 53, correct ‘evinces’ 

## Author Response

We are grateful to our reviewers for the comments and suggestions that they have offered. We hereby submit a revised version of the paper, modified in accordance with these suggestions and we are of the opinion that the paper has been strengthened by these revisions. Below, we present a point-by-point response to the reviewers' comments and we reference the sections/figures of the manuscript that have been modified or added (all modifications to the manuscript have been marked with red font).

Reviewer 1

We thank the reviewer for his evaluation of our work.

One important issue is raised regarding the influence of lower-level image differences and their impact on our results. We agree that lower-level image differences can impact results and that it is critical to consider this impact carefully. Specifically for this reason we conducted classification both within expression (i.e., by training and testing classifiers across the same images) and across expression (i.e., by training on images displaying one expression and by testing classifiers across images of the same individuals displaying a different expression). In line with the expectation that within-expression classification exhibits more sensitivity to lower-level image differences we found higher levels of classification accuracy for the former relative to the latter. Critically though, cross-expression classification, which aims to tap into expression-invariant individual face processing, yielded significant results and, further, its time course mirrored that of within-expression classification. In conjunction with the ability to map neural-based face spaces across expression, these results argue in our view for the value of higher-level expression-invariant visual information in classification and reconstruction.

More specifically, the reviewer asks whether early spikes around stimulus onset in classification/reconstruction accuracy can be ascribed to lower-level image differences across our stimuli. Three points are relevant in this respect. First, such spikes do not occur consistently across participants and across different analyses - for instance, an early spike in group-based classification accuracy only occurs briefly for classification across expression in Fig 2a but not for its within-expression counterpart or for the single-participant results shown in Fig 2b. Second, the amplitude of these spikes is visibly lower than that of later ones during intervals of interest (e.g., in the vicinity of the N170 component). And third, these spikes only achieve statistical significance in a single instance: for classification across expression in Fig 2a. However, in this specific instance we reason that it is a lot more likely that within-expression classification would capture lower-level image differences at that time, which is not the case as shown in the same figure. Further, the timing of this spike, 0 to 5ms after stimulus onset, is arguably too early to capture any stimulus-driven effect related to visual processing. Hence, in the absence of robust and consistent results we treat this specific data point as a false positive. This point is currently summarized in the manuscript: “In addition, an earlier interval of significance was found for across-expression classification between 0-5ms; however, given its very early occurrence, its reduced amplitude and the absence of its replication by within-expression classification we treat this data point as a false positive” (p. 16).

Reviewer 2

We are grateful for the comments and recommendations that our reviewer has offered.

1. The reviewer recommends that the term ‘face recognition’ be removed from the manuscript as it does not accurately reflect the topic of research. Accordingly, this term was replaced with ‘facial identity processing’ which was deemed appropriate by our reviewer.

2. It is asked whether all participants reported in the manuscripts (i.e., 13) contributed data to the study or whether any were excluded. All participants were included, as currently mentioned in the manuscript...

3. The reviewer raises an interesting issue by inquiring whether there is any effect of the database from which faces were chosen.

Given that different databases sample individuals of different nationalities (FEI - Brazilian, AR - American, Radboud - Dutch) we anticipated a possible effect of database. However, it is important to establish that the results reported in the manuscript are not solely due to such differences. To assess this possibility we examined pairwise classification results for faces extracted from the same databases, for a total of 472 pairs, and, separately, for faces extracted from different databases, for a total of 1918 pairs (p. 10). In agreement with the hypothesis above we found that classification estimates were lower for the former than the latter yet, critically, classification results were above chance in all cases. Similar results were also found by recomputing and comparing reconstruction accuracies relative to faces within the same versus different databases; however, for convenience and, also, given that classification results form the basis of all other analyses, including reconstruction, in the manuscript we focused on the impact of database on classification.

For instance, the plots below display results for temporally cumulative analyses across participants for both (a) within- and (b) cross-expression classification - analyses follow the procedure described for Fig 3b in the manuscript, except that results are collapsed across neutral and happy faces.

Parametric tests associated with these results are currently summarized in the manuscript (p. 18): “To examine possible effects of face database the analysis above was repeated while restricting pattern classification either to pairs of faces from the same database or from different databases. A two-way repeated measures analysis of variance (2 discrimination types: within/across expression x 2 pair types: within/across database) was carried out to this end - classification estimates were collapsed across neutral and happy faces given the absence of any expression effects above. This analysis revealed a main effect of discrimination types (F(1,12) =45.92, p<0.001, p2 =0.79), with higher accuracy for within than across-expression discrimination, as well as a main effect of pair types (F(1,12) =38.73, p<0.001, p2 =0.76), with higher accuracy for within than across-database classification. Critically though, all classification estimates were significantly above chance (two-tailed t-tests against 50% chance-level discrimination); mean accuracy=56.3%, t(12)=8.36,p<0.001, Cohen's d=2.32 for within-expression, within-database discrimination; mean accuracy=58.7%, t(12)=8.38, p<0.001, Cohen's d=2.32 for within-expression, across-database discrimination; mean accuracy=53.9%, t(12)=7.64 ,p<0.001, Cohen's d=2.12 for across-expression, within- database discrimination; mean accuracy =55.9%, t(12)=6.88,p<0.001, Cohen's d=1.91 for across-expression, across- database discrimination).”

Last, an incorrect reference to an additional database (i.e., FERET) was eliminated from the manuscript.

4. The reviewer asks why EEG trials have been averaged in groups of 4 to deliver observations for pattern analyses.

As currently detailed in the manuscript “this procedure was also instrumental in handling missing data following trial removal and in balancing the number of observations for pattern classification. Specifically, since it is possible that both trials associated with a given stimulus in a given block be removed (e.g., due to artifacts), averaging data across single blocks can lead to different numbers of observations for different stimuli. However, averaging data across pairs of consecutive blocks (i.e., 1-4 trials per stimulus following data removal) ensured that equal numbers of observations can be constructed for each pair of stimuli undergoing pattern classification.”

5. The reviewer asks us to specify the accuracy levels for single-participant reconstructions and also to confirm whether the “d” notation refers to Cohen's d.

Accuracy levels have been specified accordingly and corresponding references to Cohen's d have been confirmed as suggested (p. 22,23).

6. The reviewer recommends that the results of Nemrodov et al (2016) be discussed and related to the current ones.

The discussion follows this recommendation accordingly: “Thus, here we build upon previous EEG investigations of face processing and on pattern analyses of neuroimaging data to address several theoretical and methodological issues. In particular, the current work capitalizes on previous attempts at clarifying the temporal profile of individual face processing via linear classification of spatiotemporal EEG patterns across facial expression (Nemrodov et al., 2016). In agreement with this previous work we find that individual faces can be discriminated from their corresponding EEG patterns, that their time course exhibits an extended interval of significant discrimination and that multiple discrimination peaks occur, including an early one in the vicinity of the N170 component. Unlike this previous work though, which only relied on a restricted set of eight male and female faces, we find that such discrimination can be performed even with a large, homogenous set of face images controlled for low and high-level face properties (e.g., through geometrical alignment and intensity normalization of 108 Caucasian male face images). Hence, differences in discrimination onset across studies (i.e., 70ms in this previous work versus 152ms here) are likely related to reliance on idiosyncratic image differences within a small stimulus set in this previous investigation. More importantly though, not only does the current work examine the time course of individual face classification in a more reliable and thorough manner but, critically, it utilizes its outcomes for the purpose of facial feature derivation and image reconstruction.” (p. 26).

7. It is suggested that certain plots should be changed to boxplots displaying the distribution of individual results.

Following this recommendation Figures 3b and 8c, displaying the results of individual participants, have been changed to boxplots - other plots displaying permutation-based analyses for single average estimates, such as Fig 3a, are still shown as bar graphs since the distribution of a large number of permutations may be less informative and more difficult to visualize.

All other suggestions for minor modifications and corrections (missing reference, spelling, etc.) have been implemented accordingly.
